# A Review of Solar Energy Harvesting Electronic Textiles

**DOI:** 10.3390/s20205938

**Published:** 2020-10-21

**Authors:** Achala Satharasinghe, Theodore Hughes-Riley, Tilak Dias

**Affiliations:** 1 Advanced Textiles Research Group, School of Art and Design, Nottingham Trent University, Bonington Building, Dryden Street, Nottingham NG1 4GG, UK; achala.satharasinghe2016@my.ntu.ac.uk (A.S.); tilak.dias@ntu.ac.uk (T.D.); 2 MAS Innovations (pvt) Ltd., 50 Foster Lane, Colombo 10 01000, Sri Lanka

**Keywords:** electronic textiles, E-textile, smart textile, energy harvesting, photovoltaic textile, solar energy, solar cell, thin film

## Abstract

An increased use in wearable, mobile, and electronic textile sensing devices has led to a desire to keep these devices continuously powered without the need for frequent recharging or bulky energy storage. To achieve this, many have proposed integrating energy harvesting capabilities into clothing: solar energy harvesting has been one of the most investigated avenues for this due to the abundance of solar energy and maturity of photovoltaic technologies. This review provides a comprehensive, contemporary, and accessible overview of electronic textiles that are capable of harvesting solar energy. The review focusses on the suitability of the textile-based energy harvesting devices for wearable applications. While multiple methods have been employed to integrate solar energy harvesting with textiles, there are only a few examples that have led to devices with textile properties.

## 1. Introduction

For applications such as on-body sensing, electronic textiles (E-textiles) offer a distinct advantage over many other wearable and mobile devices as they are comfortable to wear close to the skin. Ideally an E-textile will possess all of the properties of a normal textile including heat and moisture transfer characteristics, shear deformability, flexibility, drape, and breathability. In addition, an E-textile should be able to survive repeated mechanical deformations, and be wash durable. While these properties are not present for all E-textiles, recent developments have led to devices with superior textile characteristics [1,2,3,4,5].

A typical sensing system consists of four main components; the sensing device, a data processing module (incorporating data acquisition, data storage or transmission, etc.), interconnections between the two, and a power supply. To date, much of the research regarding E-textile sensing systems has focused on creating textile sensors and interconnections, with data processing and power provided by a non-textile solution (i.e., Lithium Polymer batteries).

A robust and user-friendly power supply remains an unfulfilled need for many wearable E-textile devices, which is a major hurdle to the wider adoption of E-textiles [6,7]. Such a power supply is also desirable for powering other wearable and mobile devices to minimize the need for recharging. To address this requirement, there has been a significant effort in recent years to develop textile-based energy solutions, which take the form of energy storage [8,9] and energy harvesting devices [10,11], as covered by multiple excellent reviews in the literature. Of the energy harvesting technologies explored for wearables, solar energy harvesting has been one of the most investigated avenues due to the abundance of solar energy and the maturity of photovoltaic (PV) technologies [12]. While other reviews on photovoltaic textiles exist [13,14,15,16,17], this review will focus on the key textile properties of the reported photovoltaic textiles. This review is designed to be a useful resource to those working in many different areas of E-textiles, and has been written to be accessible to non-specialists. The purpose of this review is not to deeply discuss chemistries or specific material processes.

This review specifically focusses on solar E-textiles and their suitability for use in garments. As such key textile properties (bend, stretch, twist, shear, drape, appearance, softness, breathability, moisture transfer characteristics, heat transfer characteristics) and durability considerations for textiles (repeated bending cycles, repeated stretching cycles, repeated twisting cycles, wash resistance, water compatibility, sweat resistance, abrasion resistance) were the key focus. Power densities will also be discussed, as this is critical to understanding the viability of a photovoltaic textiles for a given application, and the long-term stability of the PV devices will also be reported given the limited lifetimes of some designs. The review has primarily looked at the academic literature with other sources only included to give the reader a comprehensive understanding of the area. To the knowledge of the authors this review captures all significant and closely relevant articles as of mid-2019.

To offer a holistic view of the current state-of-the-art for textile energy solutions, Section 2 briefly discusses textile energy storage and Section 3 provides an overview of other forms of textile energy harvesting. Section 4 introduces solar energy harvesting and provides important background necessary for understanding the review of the solar energy harvesting textiles, including an overview of solar cell types.

Currently a variety of different methodologies have been employed to integrate solar energy harvesting capability into textiles for wearable applications [13,14,15,16,17]. These approaches can be categorized based on either their physical structure or the photovoltaic material type. As this review is focused on the practical considerations for a wearable device the following sections will be categorized based on the construction method and architecture used for the solar E-textile. Section 5 will focus on attaching flexible solar panels onto textiles, Section 6 will discuss solar cell arrays, Section 7 will cover the application of flexible photovoltaic films and coatings onto planar textiles, Section 8 will discuss one dimensional photovoltaic structures (such as wires, fibers, and yarns), Section 9 covers textiles woven from photovoltaic fibers, and, finally, Section 10 will cover non-woven photovoltaic textiles. The final section will discuss and overview the extant photovoltaic textiles with a view to their comfort and wearability, their suitability for use with E-textile sensing systems, and offer a future perspective for this area.

## 2. Energy Storage Textiles

Battery technology is well developed and the most widely used method of supplying power to wearable devices. Most commercially available wearable systems are powered by standard solid coin cells, pouch cells, cylindrical cells, or prismatic cell batteries of an Alkaline, NiMH, Li-Ion or Lithium-Ion Polymer (LiPo) type [18]. For E-textiles, these batteries are typically attached to the surface of a garment after assembly, or embedded in a removable module, making the systems bulky and cumbersome to use [9]. Supercapacitors have also been explored for energy storage in textiles however their relatively low energy densities (compared to batteries), and high self-discharge rates limit their utility to secondary energy storage devices or for use in power regulation electronics [9].

While advancements in technology have resulted in both thin and flexible energy storage devices, attaching such devices onto a textile fabric will still have a significant impact on their textile properties, especially their heat and moisture transfer characteristics. As such, significant research has been undertaken to integrate energy storage capability within textiles, and to fabricate textile-based energy storage devices.

Both textile batteries and supercapacitor systems [8,9,19,20] have been developed with an aim to improve the appearance and comfort of the energy storage system for the wearer. Textiles and clothing provide an ideal substrate for integrating energy storage capabilities due to their relatively large surface area. Most research to date has focused on textile supercapacitors, with the first example being developed in Stanford University where a textile electrode coated in a single wall carbon nanotube ink was used to create a textile supercapacitor device [21]. Textile-based energy storage devices were reported to be superior to their polymer film and paper based counterparts, due to their flexibility and pliable nature, which prevents kinking, and their ability to recover their shape [22]. An alternative concept is to create a yarn-based supercapacitor, as first demonstrated by Bae et al. in 2011 [23].

Research has also been conducted into the creation of hybrid textile energy systems, where both energy storage and energy harvesting capabilities are combined [24], initially demonstrated by Fu et al. in 2013 where a fiber supercapacitor was combined with a triboelectric generator to allow for the energy generated from body movements to be harvested and stored [25]. This device was formed around a stainless-steel wire. Another example is a flexible ribbon with an organic photovoltaic face and flexible supercapacitor backing, which could simultaneously harvest solar energy and store electricity [26]. These ribbons were subsequently woven into a fabric. Many works regarding hybrid textile energy systems have emphasized the use of supercapacitors to either regulate the energy harvested or to store the harvested energy to minimize the frequency of recharging.

While textile energy storage remains at the forefront of research in the field of wearable energy solutions, many devices have poor durability, washability, appearance, and comfort. In addition, any devices containing corrosive liquid electrolytes pose significant safety concerns [8], this may prohibit their use in wearables.

## 3. Textile Energy Harvesting

Textile energy harvesting is an alternative or complimentary solution to energy storage systems for powering wearables and E-textiles. Energy harvesting systems collect, or harvest, ambient energy from the wearer or surroundings with thermal energy from the human body, kinetic energy due to body movements, wind energy (energy is harvested from wind that caused the fabric to move), and solar energy (photovoltaic conversion of sunlight into electricity) being amongst the most widely investigated sources of energy for wearable systems [27].

### 3.1. Thermal Energy Harvesting for Powering Wearables

Thermal energy can be harvested from the wearer using a thermoelectric (TE) generator. These generators are semiconductor devices that generate an electric current when exposed to a temperature gradient between two surfaces (via the Seebeck effect). Electrical energy can therefore be harvested by taking advantage of the temperature gradient between the human body and environment, with the electrical power generated being dependent on this temperature gradient. This means that the greater the temperature difference between the wearer’s skin and the environment, the more energy that can be harvested. The literature reports a number of attempts to develop wearable thermoelectric generators [28,29,30,31,32,33,34,35,36,37].

The method employed by Leonov [35] was to attach rigid thermopiles onto a textile (for example a shirt), while Seeberg et al. [36] and Kim et al. [37] realized textile based TE generator systems by using screen-printing techniques on textile substrates (woven cotton and a glass fabric, respectively). Kim et al. reported the resulting TE generator to be both lightweight and flexible.

Moreover, it is to be noted that to achieve power conversion efficiencies greater than 1%, the devices need to be exposed to temperature gradients in excess of 20 K which are not practical for most wearable applications. For example, the TE generator system by Kim et al. had a power density of 38 Wm^−2^ but only with a temperature gradient of 50 K [37].

Typically TE generators, including for wearable applications, are fabricated using conventional thermo-electric materials such as Bi_2_Te_3_ and PbTe which are brittle, toxic, heavy, and therefore undesirable to use for wearable applications [1,29]. While flexible TE generators can be achieved these do not exhibit other typical textile properties such as breathability, or drape. This will affect the comfort of the wearer when using the device.

An alternative means of harvesting thermal energy are pyroelectric generators which rely on temperature fluctuations (change in temperature over time) caused by thermal diffusion [38]; however, low levels of skin temperature fluctuation make this technology unsuitable for wearable applications [29].

### 3.2. Piezoelectric Generators for Powering Wearables

Piezoelectric generators can be used to harvest energy from human motion. These generators incorporate a piezoelectric material which will generate an electron flow when subjected to mechanical strain by either being compressed or tensioned. For wearable applications, piezoelectric generators are typically fixed onto regions on the body where large dynamic compressive forces (directly compressing the material) or tensile forces (indirectly stretching strands of the fibers) due to motion are generated.

A number of textile based piezoelectric generators have been reported in the literature [39,40,41,42,43,44,45,46,47,48,49]. Different types of piezoelectric strands have been produced by wrapping or twisting piezoelectric fibers, nanofibers or yarns, and through different fiber spinning techniques, such as electrospinning and melt spinning a bi-component fiber made with a poly(vinylidene fluoride) sheath and a conductive high density polyethylene/carbon black core. These piezoelectric fibers and strands have also been converted into textiles leading to woven, knitted, braided, non-woven, and spacer fabric structures [46,48,49], which may offer useful textile energy harvesting solutions. One of the highest reported power densities for piezoelectric fabrics was reported by Qin et al. for a twisted microfiber-TiO_2_ nanowire hybrid yarn where a power density of 80 mW/m^−2^ was achieved [50].

### 3.3. Triboelectric Generators for Powering Wearables

The triboelectric effect sees a material become electrically charged after it is separated from another material; contact-induced build-up of static electricity is an example of the triboelectric effect. The contact and separation between the materials can occur in a number of ways (i.e., sliding) and triboelectric generators can be used to harness energy from vibrations or frictional forces generated by either human motion or the wind. Triboelectric generators can generate high power densities, and high voltages of up to 1200 V [51]. These high power densities are instantaneous, generating a high electrical potential difference between the triboelectric surfaces with small pulses of current. A variety of textile and wearable triboelectric nano-generators have been reported in the literature in recent years [52,53,54,55,56,57,58,59,60,61], with power densities as high as 500 W/m^2^ being reported [61].

Despite the high voltages and power densities achievable with triboelectric generators, these systems are highly volatile and the total energy generated over a time interval is unpredictable. Although the Triboelectric generators have some inherent energy storage capability in the form of dielectric capacitors [62,63] this internal capacitance is not sufficient to regulate the power output. Therefore, triboelectric generators require sophisticated energy management systems to convert and regulate the power before being used to power devices [64].

### 3.4. Electromagnetic Induction-Based Energy Harvesters for Powering Wearables

Electromagnetic induction occurs when a conductive coil is placed within a varying magnetic field generating an electric current in the coil, and therefore this technique can be used to harvest energy from motion. Electromagnetic induction has been investigated for powering wearable devices [65,66,67,68,69], especially for shoes. An electromagnetic induction-based energy harvester normally has a permanent magnet at its core which is free to move inside of a tubular structure onto which conductive induction coils are mounted. The reported textile-based devices of this kind are typically heavy, rigid and bulky—this makes them unsuitable for most textile-based solutions. As with piezoelectric generators and triboelectric generators, the power output of electromagnetic inductors is dependent on motion and can therefore be inconsistent.

## 4. Solar Energy Harvesting

**Figure 1:**
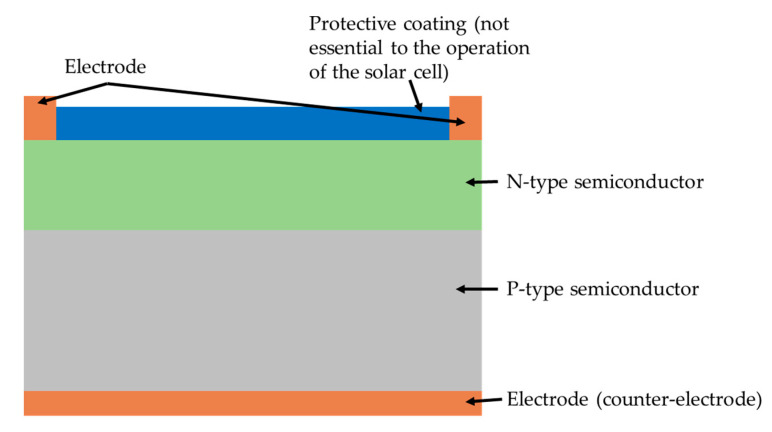
Simple schematic of a typical solar cell comprising of two semiconductor materials (P-type and N-type) and two electrodes.

Solar energy can be harnessed by irradiating sunlight upon semiconductor materials that can release free electrons in order to generate an electron flow. This effect was first observed by French physicist A. E. Becquerel in 1839, and is defined as the photoelectric effect [70]. A typical solar cell comprises of two semiconductor materials (P-type and N-type) and two electrodes (often referred to as the electrode and counter-electrode), as shown in Figure 1.

The energy harvested by a solar cell is typically characterized using two parameters, fill factor (FF) and power conversion efficiency (PCE).

**Figure 2:**
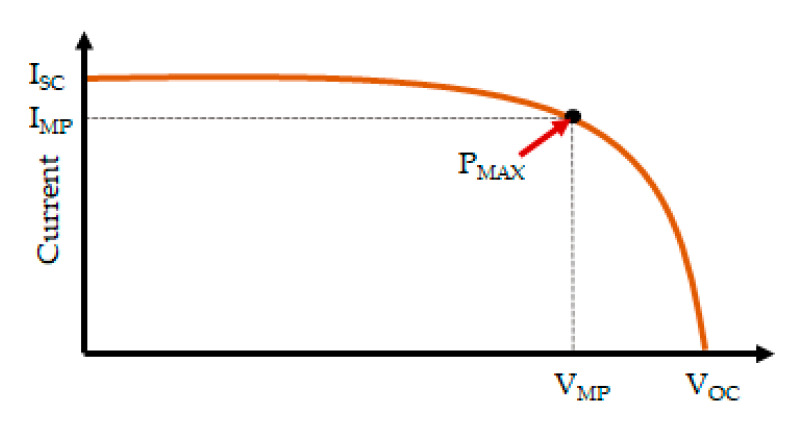
Diagram showing a typical current-voltage curve for a solar cell. The point where the maximum power is achieved has been annotated on the figure. This figure has been drawn and is not generated from collected data.

FF is a characteristic parameter for a given cell type and indicates the performance of the cell when the maximum power is drawn. It is defined by Equation (1) below.

(1) $$\mathrm{FF}=\frac{V_{MP}\times I_{MP}}{V_{OC}\times I_{SC}}$$

where *V_MP_* and *I_MP_* are the maximum voltage generated and maximum current generated (respectively) at the point where the maximum power (*P_MAX_*) is generated. *V_OC_* (the open circuit voltage) is the voltage across the solar cell when no connection is made between the terminals (zero current is drawn from the cell), which is the maximum possible voltage. *I_SC_* is the short circuit current which represents the current flow through a zero-resistance conductor when connected between the terminals of the solar cell, which is the maximum possible current. A current-voltage characteristic curve showing the behavior of a solar cell is shown in Figure 2.

The power conversion efficiency (PCE) of a solar cell is the percentage of the incident light energy reaching the solar cell (*P_IN_*) converted into electric energy by the solar cell (Equation (2)).

(2) $$\mathrm{PCE}=\frac{V_{MP}*I_{MP}}{P_{IN}}\times 100\%=\frac{FF*V_{OC}*I_{SC}}{P_{IN}}\times 100\%$$

Both the *FF* and PCE will be dependent on the type of semiconductor used in the solar cell.

Solar cells can be categorized into three generations, mainly based on semiconductor material combinations. The first generation of solar cells were the mono-crystalline/multi-crystalline single junction type, such as the crystalline silicon (c-Si) solar cell, which still dominates the market today. First-generation solar cells are typically rigid and brittle, and therefore need to be mounted on rigid frames to protect the cells from breaking. Second-generation solar cells were produced as thin films to reduce manufacturing costs [71], with the trade-off being lower efficiency levels compared to single crystal types (with the exception of more expensive gallium arsenide (GaAs) cells). The third-generation of solar cell technologies include multi-junction cells, organic photovoltaic (OPV) cells, and hybrid solar cells. Hybrid solar cells, namely dye-sensitized solar cells (DSSC) and perovskite solar cells, employ inorganic and organic semiconductor material combinations. Most of these third-generation cell types are yet to make a significant entry into large scale commercial applications due to the challenges faced in scaling up of production, durability, or cost. Despite this, multi-junction solar cells have successfully managed to enter extra-terrestrial and concentrated photovoltaics markets due to their unparalleled conversion efficiencies (PCE = 38.8 ± 1.2% [72]); however, their volumes remain small due to their high cost. In recent years, the organic and hybrid cell concepts have shown great promise in achieving improvements in conversion efficiency and durability, especially for application demanding mechanical flexibility [71].

## 5. Integrating Flexible Solar Panels with Textiles

The simplest method of adding solar energy harvesting capability to a textile is to superficially attach flexible solar panels onto the surface of a fabric: This method has been employed to develop many consumer products [14,73], including a plethora of solar backpacks available on the market. This includes the Kingstons Beam Backpack (Kingstons, Guangzhou, China) which has an integrated flexible thin film copper indium gallium selenide (CIGS) solar cell on its outer surface (produced by MiaSolé, Santa Clara, CA, USA) [74] with a conversion efficiency of 19.2% [75]. Similarly the Zenith Solar Backpack (by Sunslice) uses a flexible CIGS solar panel (with a 15% PCE) attached to the backpack’s outer face [76]. The use of CIGS cells for these products is likely due to the flexibility that can be achieved when fabricated on a plastic backing and their reasonable power conversion efficiencies. It should be noted that most CIGS are produced using a glass substrate, in which case these cells would not be flexible.

Flexible solar panels have also been attached onto the surface of apparel and early examples include the Maier Sports’ prototype of a winter outdoor jacket, which was first presented in Munich at the International Trade Fair for Sports Equipment and Fashion 2006. The jacket incorporated nine amorphous Si solar modules from AkzoNobel (Amsterdam, Netherlands) and could generate a *P_MAX_* of 2.5 W under full sun conditions [13]. Amorphous silicon (a-Si) is an alternative thin film solar cell technology which can achieve flexibility by creating the solar cells using a flexible substrate. It is unclear from the available literature whether the solar cells used for this jacket were definitely flexible, however it seems likely as if flexibility was not desired c-Si cells would have likely been used to achieve a higher PCE. Tommy Hilfiger’s solar powered jacket from 2014 used a similar concept [77], and comprised flexible amorphous silicon solar cells developed by Pvilion (New York, NY, USA); these solar cells were flexible [78]. The fashion designer and researcher Pauline van Dongen developed a collection of designer wear where thin film solar cells were attached onto garments [79,80,81] including: the ‘Wearable Solar Dress’ from 2013, ‘The Solar Shirt’ from 2014 which can generate 1 W under direct sunlight; the wearable solar dress, also from 2014; ‘The Solar Parka’ from 2015; ‘The Solar Windbreaker’ from 2016. The ‘Wearable Solar Dress’ and’ The Solar Shirt’ both utilized flexible solar cells of an unreported type [81]. These cells were flexible, but not foldable, so multiple separate solar cells were incorporated into the garment, as opposed to a single large cell, to allow for greater movement between the cells. Despite this the ‘Solar Dress’ was reported to limit movement and the wiring could be felt. ‘The Solar Shirt’ used printed electronic interconnections between the cells, and the garment was reported as being more flexible.

Garments with attached flexible solar cells effect the appearance, and may affect the comfort of the wearer, which will likely limit their application to outerwear and futuristic fashion prototypes. While solar cells can be made water compatible it is unknown how garments made using flexible solar cells attached to the surface would cope with the relatively severe conditions of a domestic machine wash. In order to achieve satisfactory levels of wearability (softness and comfort) and washability, conformal (drapeable and shear deformable) PV devices with the structural (hierarchical and porous) features inherent to a textile structure are necessary.

## 6. Solar Cell Arrays

A similar concept to attaching flexible solar cells onto textiles is to exploit rigid inorganic semiconductor PV materials by mechanically and electrically linking the inorganic solar cells together with flexible materials to create quasi-flexible structures. A number of such embodiments for wearable applications have been proposed in patents and patent applications in the form of solar jackets or suites [82,83,84], non-garment based solar textiles [85,86,87,88,89,90], and more generally flexible interconnected solar cell networks based on fabrics [91,92,93,94,95,96,97]. The products and processes disclosed in these patents appear to be of limited use for practical wearable systems, due to an inadequate level of flexibility, lack of shear behaviour, and unsuitability for machine washing. In addition, the appearance rendered by these fabric attached devices were not desirable for the use on regular clothing. However, without examples of completed textiles, it is difficult to conclusively comment on these concepts.

**Figure 3:**
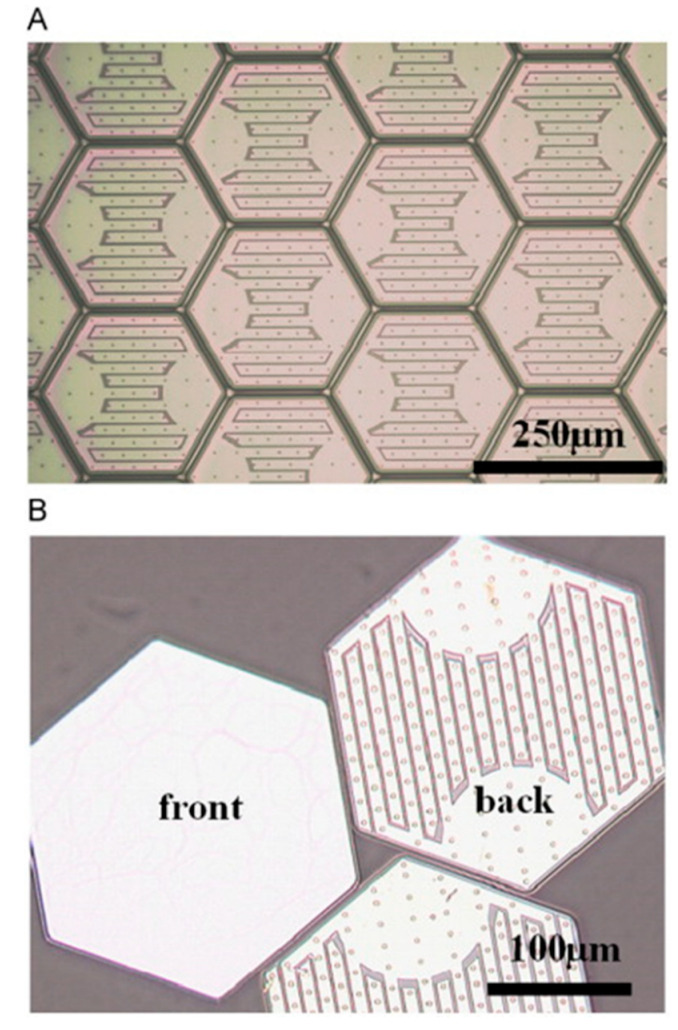
Microscope image of micro-structured PV cells developed by Sandia Laboratories. Reprinted from [98] with permission from Elsevier. (**A**) An array of cells attached to the wafer (**B**) The front and back the cells.

Sandia National Laboratories have worked with micro-scale inorganic photovoltaic cells for concentrator photovoltaic applications, which were also explored for flexible solar arrays as reported in 2011 [98] (see Figure 3). This PV technology has been demonstrated in flexible moldable concentrator PV arrays, as shown in 2015 [99], as well as flexible arrays of closely packed cell arrays mounted on a flexible substrate as shown in 2017 [100]. The bespoke small-scale PV cells have a hexagonal shape, with back contacts, and typically have a thickness of 14 µm and a width from 250–500 µm. With an optimized cell design a PCE of 14.9% has been achieved. This innovation, while potentially useful for E-textile applications, has not been presented for this use. As a result information on other properties that would be needed for a wearable E-textile is not present in the literature; however, given the dense structure of the solar cell array if worn, the array would not provide the wearer with a normal textile feel as the array is not porous or soft, and lacks a normal textile appearance. It is unclear if the array would be sufficiently durable to survive machine washing.

**Figure 4:**
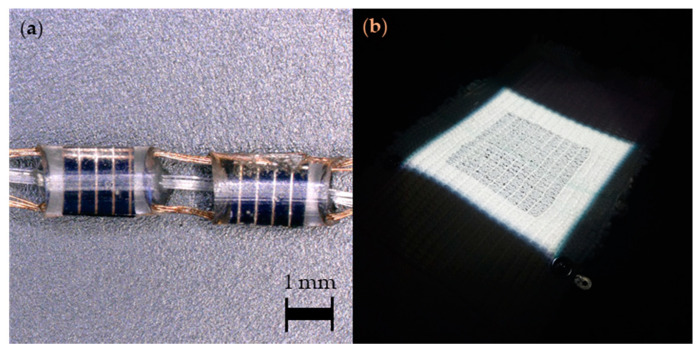
(**a**) Rigid solar cells soldered onto flexible copper wires. The filaments would subsquently be covered with textile fibers. (**b**) A woven solar fabric.

In 2019, researchers in the Advanced Textiles Research Group at Nottingham Trent Unviersity demonstrated a method of embedding arrays of small-scale solar cells (1.5 mm × 3.0 mm × 0.2 mm) within textile yarns, which were subsequently woven into fabrics (Figure 4). The rigid solar cells were linked using flexible copper wires producing fabrics with normal textile properties. This design has been shown to be bendable, can undergo shear deformation, and is drapeable; however, these characteristics have not been fully quantified in the literature. Fabrics were also wash tested using standard domestic laundering and retained ~90% of their original power output after 15 machine wash cycles. A PV fabric with a 44.5 mm × 45.5 mm active area generated ~2.15 mW/cm^2^ under one sun illumination (2.15% PCE), which was demonstrated to charge a mobile phone and fitness tracker [101]. Compatibility with water was also demonstated. The funtionality of the solar cell embedded fabrics after 6000 abrasion cycles showed a 5.6% reduction in *I_SC_*.

## 7. Applying Flexible Photovoltaic Films and Coatings onto Planar Textiles

**Figure 5:**
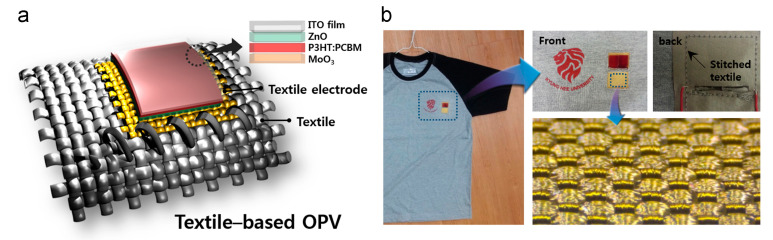
A textile based OPV developed by Lee et al. Reprinted from [105] with permission from Elsevier. (**a**) Schematic illustration of the textile OPV. (**b**) Photographs of the textile OPV integrated with clothing.

Printing, laminating or coating [102] organic photovoltaics (OPV) [103,104,105], hybrid photovoltaics (such as DSSCs [106,107] and perovskite solar cells [108,109]) onto planar textiles has been widely investigated for textile-based PVs (an example of a film-based textile OPV is shown in Figure 5).

### 7.1. Organic Photovoltaic Films and Coatings

In 2006 Krebs et al. [103] reported one of the first printed PVs intended for use with textile fabrics. Here, organic photovoltaic devices were fabricated onto non-transparent polyethylene terephthalate (PET) or Polyethylene (PE) tapes, where two strategies of fabric integration were explored. The first method saw the PET tapes coated with organic PV materials before being laminated on to a suitably transparent textile fabric, which resulted in a somewhat stiff device. In the second method, PE tapes were first laminated onto the fabric prior to the application of the PV coatings and electrodes, making a more flexible device. The PCE of these textile coated PVs was 1.4 × 10^−3^%, and they had a fill factor of 25%. A substantial degradation in this performance was observed within two hours of direct exposure to light (one Sun intensity), which was mainly attributed to the instability of photovoltaic material under sunlight. Similar work was presented by Bedeloglu et al. in 2009 [110], where polymer PV coatings were used on six different substrates (polypropylene based and ITO/glass based). A maximum PCE of ~0.37% was achieved for the ITO/glass-based substrate. Bedeloglu et al. also realized a 0.33% PCE for a flexible nano-silver coated polypropylene (PP) tape that could be laminated onto textiles.

**Figure 6:**
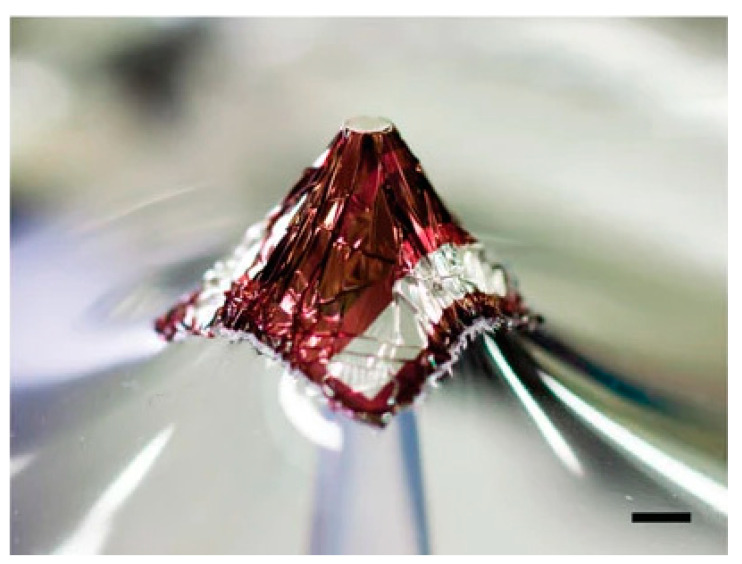
The thin polymer PET foil based solar cell attatched to an elastomeric support developed by Kaltenbrunner et al. This image shows the three-diamentional deformablity of the structure when deformed by applying pressure from a 1.5 mm diameter tube. Taken from [112]; this image is licensed under a Creative Commons Attribution-NonCommercial-Share Alike 3.0 Unported License.

In order to make flexible PV films more compliant for wearable applications, in 2011, Lipomi et al. [111] devised a stretchable polymer solar cell by spin coating a pre-strained polydimethyl siloxsane (PDMS) film with polymeric PV materials. These stretchable films achieved a PCE of 2%. These devices were tested under different levels of mechanical loading (stretching), observing that the PV characteristics of the devices had little dependency on the strain applied. This strategy of coating pre-strained PDMS, or similar elastic films, was adopted widely in subsequent studies to achieve stretchable PV films. In 2012, Kaltenbrunner et al. prepared thin polymer PV films on PET foils which exhibited a PCE of 4% [112]. It was possible to attach these thin films onto pre-stretched elastomeric films to realize flexible, stretchable PV films. The films exhibited three-diamentional deformability (as demonstrated in Figure 6). Pre-stretched films were tested under extreme mechanical deformation, being compressed by 80% and still functioning (alblight with the PCE reducing to ~0.25 of its intial value), and showing no deterioration in performance when re-stretched. Cyclic compression and stretching tests to 50% compression were also shown, with a PCE of 73% being maintained after 22 cycles.

Kylberg et al. developed organic PV coatings on woven structures that were made with a combination of metal and polymeric mono-filaments in 2010 [113]. One side of the woven structure was coated with a transparent PET filler material (this side was used as the active side of the cell), with the back electrodes coated on the other side of the woven structure. This construction resulted in a flexible polymeric film with a PCE of 2.2%.

An organic photovoltaic cell where a fabric electrode was prepared by weaving PE multifilament yarns coated with conductive metal multilayers was proposed in 2014 [105] (shown in Figure 5). The authors presented a construction where at least part of the solar cell was a textile structure. This OPV could be stitched and was integrated onto a T-shirt. These solar cells had a PCE of 1.8%.

In 2016, Arumugam et al. realized a fully spray coated OPV on a polyester cotton blended plain woven fabric by smoothening the fabric surface using a screen printed interface layer [114]. This construction only yielded a PCE of 0.02%. While flexible, bending and cyclic bending tests resulted in the devices losing their functionality.

An organic PV film was presented by Jinno et al. in 2017 which exhibited a high PCE of 7.9% [104]. The film was developed for wearable applications and was both waterproof and washable. The devices were stretchable and flexible. Images of the device show that it was highly deformable and may exhibit drape characteristic (however, this was not claimed by the authors). Both compression and cyclic compression were examined, with the (single side coated) device retaining 99% of its original PCE after 20 cycles at 43% compression. The washability of the OPV film was demonstrated by placing the OPV in a detergent-water mix which was stirred for 5 min. Various experiments were conducted to demonstrate the water stability of the device. To improve compatibility with water and achieve stretchability, the OPV film was sandwiched between two layers of stretched acrylic elastomer. Further tests were conducted by dipping both the OPV film and sandwiched devices into distilled water for 120 min, which showed ~20% and ~5% reductions in PCE, respectively. The PCE of the device was reported to reduce by 46% after 1000 h.

A study on textile OPVs from 2019 used a SiO_2_-polymer composite capping-layer as a protective encapsulation [115]. The wash durability of these OPV devices was tested by stirring them in 2% detergent solution inside of a beaker for 10 min after subjecting the device to 1000 bending cycles (3 mm bend radius). This test was repeated up to 20 times for thirty days and the OPV devices showed no significant change in performance after this time. These devices also generated an impressive PCE of 7.26%. This construction, however, was not stretchable, although it could undergo thousands of bending cycles without a deterioration in performance.

### 7.2. Dye Sensitized Solar Cell Films and Coatings

One of the first studies on textile based DSSCs was presented by Du et al. in 2013 [102] who created DSSCs with an iodide/triiodide (I−/I3−) electrolyte (liquid electrolyte); these flexible devices demonstrated a PCE of 3.93%; however, their long-term stability was not evaluated. A flexible cotton fabric based DSSC was presented in 2014 by Xu et al. and yielded a PCE of 3.3% [116]. This flexible device used a liquid electrolyte. The PCE was further enhanced up to 3.83% by the same research team in 2016 [117].

**Figure 7:**
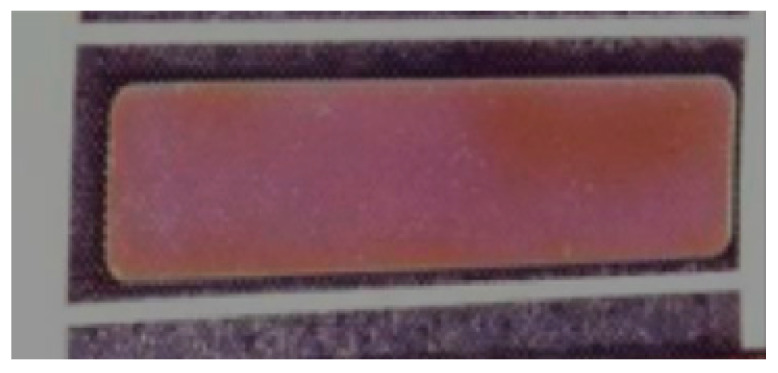
Photograph of a textile-based dye sensitized solar cell developed by Opwis et al. Adapted from [119]; this image is licensed under a Creative Commons Attribution 4.0 International License (CC BY 4.0).

Yun et al. prepared DSSCs on metal wire woven structures, without using transparent conducting oxides, and a liquid electrolyte in 2016, which generated a PCE of 4.16% [118]. This flexible device was tested when bent at different radiuses of curvature and was seen to maintain 80% of its performance after 2000 bending cycles. The same year Opwis et al. realized a flexible DSSC on a polyamide-coated glass woven fabric where a liquid electrolyte was used, which generated a PCE of 1.1% [119] (see Figure 7); these had a stable performance for at least seven weeks and showed improved efficiency at lower incident light intensities. The study also reported the performance of the DSSC at different operating temperatures.

**Figure 8:**
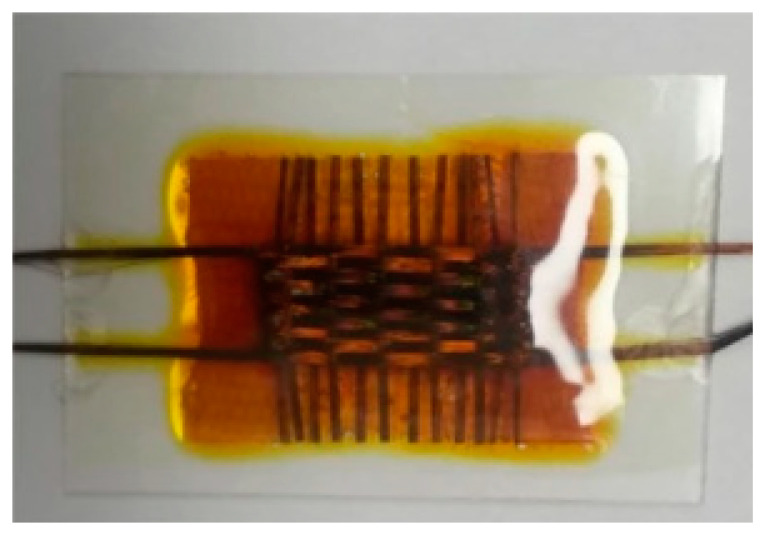
3D textile DSSC developed by Yun et al. Adapted from [120]; this image is licensed under a Creative Commons Attribution 4.0 International License (CC BY 4.0).

A DSSC was developed on woven polyester cotton blended fabric by Liu et al. in 2018, by screen printing an interface layer onto the fabric before coating it with a silver electrode, with the resultant DSSCs exhibiting a PCE of 2.78% [106]. This approach was similar to how Arumugam et al. prepared the fully spray coated textile based OPVs [114]. This device was not flexible, using a rigid platinum coated FTO glass for one electrode. A liquid electrolyte was used with this design. In 2019, Yun et al. developed a textile DSSC based on a three-dimensional structure where an electrolyte-infused woven glass-fiber spacer fabric was sandwiched between two stainless steel woven fabric electrodes coated with active materials [120] (see Figure 8). The final structure was encased inside of a polyester film pouch which was filled with a liquid electrolyte. This design of textile DSSC generated a PCE of 1.7%. The devices were tested when bent to different radiuses of curvature proving its flexibility. Cyclic bending tests demonstrated a reduction in efficiency of 40% after 1000 bending cycles, with no further reduction observed after 2000 bend cycles. By using a non-volatile electrolyte, the DSSC saw a slight increase in specific power after one day, with specific power remained stable for the following six days.

Building on work from 2017 [121], in 2019, Song et al. reported a flexible DSSC photovoltaic textile, created using a quasi-solid electrolyte, with a power conversion efficiency of 5.08% that was achieved by attaching a DSSC film onto a textile using an epoxy [122]. The work demonstrated counter-electrode designs that could be twisted, had good mechanical strength, and that could be bent multiple times, leading to a final DSSC textile that was flexible.

### 7.3. Other Types of Solar Cell Films and Coatings

Solar energy harvesting textiles using other types of coating or film technology have also been developed.

**Figure 9:**
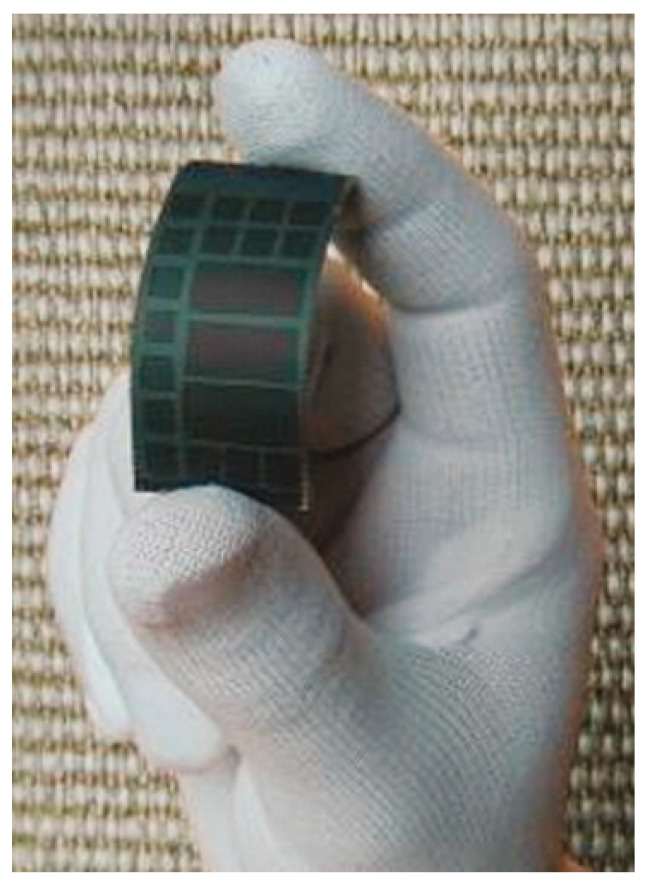
Photograph of the flexible copper indium gallium selenide thin film solar cell on glass fabric developed by Knittel et al. Reprinted from [123] with permission from Wiley.

A flexible CIGS thin film coating was presented on glass-fiber-woven fabrics by Knittel et al. in 2009 (shown in Figure 9), which achieved an efficency of 8.0% [123].

**Figure 10:**
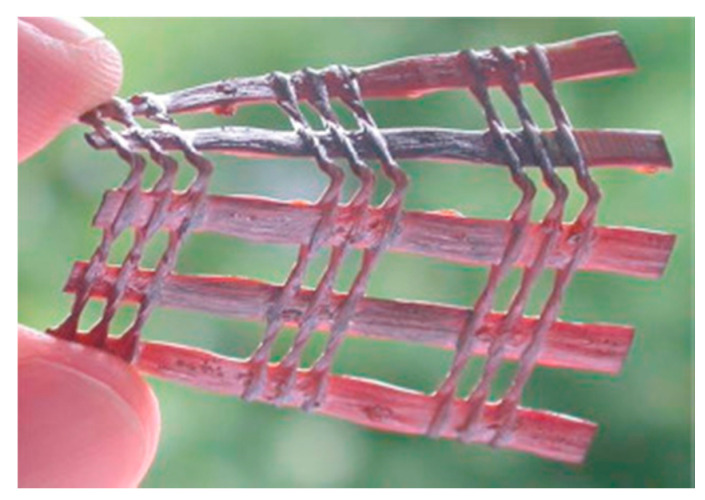
Thin film solar cell on glass fiber fabric developed by Plentz et al. Reprinted from [124] with permission from Elsevier.

A hydrogenated amorphous Si thin-film solar cell was prepared on a glass fiber fabric by Plentz et al. in 2016. The device (see Figure 10) realized a PCE of 1.41%; however, this approach was not suitable for applying PV functionality directly onto standard textiles substrates such as PA or PET due to the high processing temperatures necessary [124].

In 2017 Lam et al. reported a flexible textile-based perovskite PV laminate using a SnO_2_/PCBM active layer and an elastomeric encapsulation covering the fabric and active layers, which demonstrated a PCE of 15% [108]. The work claimed that the ductility of the encapsulating elastomer provided good flexibility and the potential for washability; however, experimental evidence to prove these claims was not presented. The stabilty of the device was evaluated seeing that it was able to maintain 70% of its initial PCE after 425 h. Water compatability was also shown, with the device showing little deterioration in performance after 35 min submerged in water.

In 2018, Jung et al. reported flexible perovskite solar cells where a polyurethane coating on the textile fabric enabled low-temperature solution processing, resulting in solar cells that achieved a PCE of 5.7% [109]. The device maintained 83% of its PCE after 300 h.

### 7.4. Discussion of PV Films and Coating Applied to Textiles

The key information regarding photovoltatic films and coatings applied to textiles are summarised in Table 1.

Table 1 clearly shows that flexibility is the property that is most reported, with many works also presenting data testing the PV device after multiple bending cycles. Some stretchable devices have also been demonstrated. The OPV devices by Kaltenbrunner et al. and Jinno et al. were both highly deformable [104,112], with the device by Kaltenbrunner et al. showing 3D deformation (as shown in Figure 6). Only Lam et al. and Jinno et al. demonstrated compatibility with water for their devices [104,108]. Jinno et al. also showed that their device was washable under mild conditions [104]. While the device proposed by Lam et al. was perovskite-based, water compatibility was achieved by using an encapsulation [108]. This also significantly extended the lifetime of the cell under ambient conditions, as a version of the cell without the encapsulation degraded significantly after 100 h due to Perovskite’s instability to moisture and oxygen. The stability of perovskite solar cells is a known limitation to the technology. Stabilities of other devices were largely unreported, however Opwis et al. and Jeong et al. showed that their devices had good stability over many weeks [115,119]. The PCE of the reported devices ranged significantly with OPV devices with a maximum PCE of 7.9% [104], DSSCs with a maximum PCE of 5.08% [122], and Perovskite-based cells with a PCE as high as 15% presented [108].

Without further testing and details on durabilty, it is difficult to fully comment on the suitabilty of these materials for textile garment applications. The general suitabilty of these devices may be limited by two factors. These PV systems significantly alter the color and fibrous surface morphology of textile fabrics. For example, most PV films have a smooth glossy surface with a rubbery or foil-like texture, and the colors are typically limited to the inherent colors of the photoactive or electrode material. Furthermore, these devices require coatings with excellent barrier properties to achieve long term stability and compatibility with water. This results in an E-textile that is non-porous and non-permeable, inhibiting the breathability and moisture transfer characteristics of the fabric, which will significantly alter the feel to the wearer. This will also have some influence on the textile’s heat transfer characteristics. This will not be the case for the textile proposed by Plentz et al. as shown in Figure 10, as this structure has large pores in it (however, the structure lacks the appearance of a normal textile) [124].

## 8. Photovoltaic Wires, Fibers, and Yarns

The potential of developing one-dimensional (1D) PV devices in the form of fibers, yarns, wires, or tapes for fabricating planar textile PV devices has been explored in the literature. Two main routes have been investigated for creating 1D PV devices. Initially, metal wires, carbon-based fibers or conductive polymer filaments were employed as the core of the device, with the other layers built on top to create the PV fibers. The other method presented in the literature utilized conventional polymeric textile fibers as the base material onto which the 1D PV devices were built. The latter approach is more desirable for retaining the textile features of the resultant planar structure, while the former allows for a simpler fabrication process and better device performance (in terms of PCE).

### 8.1. 1D Photovoltaics Devices Using Non-Polymeric Fibers or Wires as a Core

The use of metal wires or carbon based fibers as the core material to craft PV fibers, where the conductive core was coated with various photovoltaic material combinations to create organic, hybrid, or inorganic coaxial-fiber shaped solar cells, has been investigated in the literature.

Hybrid PV (predominantly DSSCs) technologies have dominated the research on PV fibers. The liquid or gel electrolytes used in most DSSCs are conformable and allow the PV fibers to deform while maintaining the integrity of the PV structure. However, DSSCs can suffer performance degradation caused by leakage of the electrolytes due to the deficiencies of the encapsulation.

#### 8.1.1. 1D DSSCs Using Non-Polymeric Fibers or Wires as a Core

Early studies of 1D DSSCs from 2008 showed limited flexibility and mechanical robustness due to the capillary tube encasements being employed, and a poor adhesion between the Ti wire electrode and TiO_2_ nano coating [125]. Several reports have seen the metal wire counter-electrodes being replaced by transparent conductive metal oxide (e.g., ITO) capillary tubes; these tubes also served the purpose of encapsulating the device, however, they make the device stiff and rigid which is undesirable for a textile application. In 2011, Lv et al. reported a DSSC wire with a twisted working electrode/counter-electrode configuration encapsulated by an electrolyte filled capillary tube, which generated a PCE of 5.41% [126]. This work used a rigid glass capillary tube; however, it is claimed that a flexible device could be created if the tube was replaced by a flexible polymer. Yang et al. managed to realize a PCE of 8.45% in 2013 with a Ti/TiO_2_/Pt working electrode by replacing the Pt counter electrode with a Pt nanoparticle modified graphene oxide (GO) fiber [127]. High flexibility, good mechanichical strength, and good stability were claimed. A multi-working electrode structure with six Ti/TiO_2_ working electrodes assembled around a Pt counter electrode was proposed by Liang et al. in 2015. The assembly was inserted into a flexible plastic capillary tube filled with a liquid electrolyte. This multi-working electrode structure achieved a PCE of 9.1% and was highly flexible [128]. Bend testing to different bending angles showed a good performance when the device was bent, maintaining a PCE of 8.5% with a 180° bending angle.

**Figure 11:**
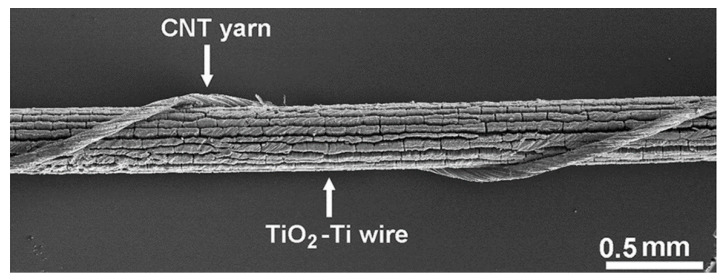
SEM image of a TiO_2_-Ti wire primary electrode diameter twisted around a carbon nanotube (CNT) yarn counter electrode. Reprinted (adapted) with permission from [133]. Copyright 2012 American Chemical Society.

DSSC PV fibers made with carbon-nanotube (CNT) working electrodes and counter electrodes have also been presented in the literature [129,130,131], which resulted in metal free PV fiber devices, improving the flexibility and ability for the fibers to be woven. This includes a DSSC by Chen et al. from 2012 which used two CNT fibers and a liquid electrolyte, creating a cell with a PCE of 2.94%. The resultant fiber was reported to be both flexible and stable. The cell was also shown woven into a textile structure using other CNT fibers [129]. In 2012, Chen et al. also presented a DSSC that could achieve a PCE of 4.6% [132]. Velten et al. developed a DSSC in 2013, also using CNT fiber electrodes and a liquid electrolyte, and achieved cells with PCEs as high as 3.4% [130]. It was claimed that these fibers were weavable; however, this was not shown in the work. Zhang et al. proposed a modified CNT based DSSC fiber which used a liquid electrolyte in 2012, which could yield a PCE as high as 4.85%, see Figure 11 [133]. The cell was flexible, showing little variation in performance when bent upto 180°. The cell was also tested after 500 bending cycles of 180° showing some reduction in performance. Stability to air was shown over 20 min with a drop in effeiency from 3.43% to 3.16% reported. The authors note that an appropriate encapsulation is needed to ensure stability on a longer timescale.

Yan et al. presented a solid electrolyte-based twisted DSSC in 2014 which generated a PCE of 6.24% [131]. With a mirror placed behind the cell, a PCE of ~7.39% was reported. The cell was flexible and showed little change in performance when bent up to 180°. The cell was also shown to have a stable perfromance when tested over a range of operating temperatures (−10–48 °C). The stability of this type of cell to multiple irradiation cycles (100), and exposed to atmosphereic conditions for up to 50 h, was shown by the group in an earlier work in 2013 [134]. This particular cell had a PCE of 2.57% and also showed a stable performance over a range of temperatures (0–45 °C) and bending angles (30–330°).

In 2018, Fu et al. reported DSSC type PV fibers with a PCE of 10%, the highest to date. The PV fibers were created using a core-sheath twisted CNT fiber counter electrode modified with Pt [135]. The DSSC fibers showed >80% of its original efficiency after 2000 bending cycles with a 90° bend. The long-term stability of the device was not reported. The researchers hand stitched the PV fibers onto a T-shirt and demonstrate their power generation capability by connecting the PV fibers to a commercial pedometer. This is a rare example of a developed solar E-textile being demonstrated to sucessfully power a sensing system.

#### 8.1.2. 1D Perovskite Solar Cells Using Non-Polymeric Fibers or Wires as a Core

Perovskite-based 1D PV fibers have also been developed. These solar cells used a similar working electrode and counter electrode configuration to the DSSCs while the dye-electrolyte combination of a DSSC was replaced by a perovskite active material, enabling an all solid-state PV fiber.

One of the first perovskite PV fibers intended for use in fabrics was reported by Qiu et al. in 2014 [136], with this construction managing to generate a PCE of 3.3%. This flexible device was shown to maintian 95% of its original efficency after 50 bending cycles. The device was also shown woven into a textile. He et al. followed a similar approach in 2015, with their highly-deformable PV fiber generating a PCE of 2.61% [137]. The PV fiber showed a 7% change in performance after 200 bending cycles where a 30° bending angle was used. Hu et al. reported a perovskite PV yarn in 2016 which had a PCE of 5.3% [138].

In 2015, Li et al. presented a double twisted perovskite yarn, which was developed by coating one CNT fiber bundle with TiO_2_/sensitizer/hole transport layers and twisting it with another CNT fiber bundle [139]. This PV fiber realized a maximum PCE of 3.03% and was stable after 96 h in ambient condition (if sealled with a polymer layer), and 1000 bending cycles. The angle by which the fiber was bent during each cycle was not presented in the work.

#### 8.1.3. 1D Coaxial Devices Using Non-Polymeric Fibers or Wires as a Core

**Figure 12:**
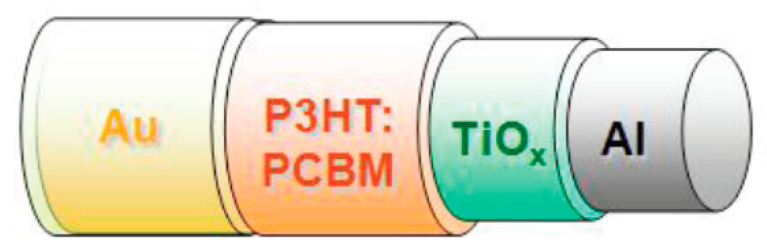
Schematic of a simple metal core based organic PV fiber. Reprinted from [140] with permission from Elsevier. This simple design has conducting inner and outer electrodes (Au and Al) and active layers (TiOx and P3HT:PCBM) forming the solar cell. This schematic provides a good example of a coaxial type solar cell, where the cell is built from multiple layers covering a core material.

Coaxial fiber PV devices have also been created using OPVs (as shown in Figure 11) [140,141,142] inorganic PVs [143] or thin-film PVs [144]; however, these have not made significant advancements in performance and have not been frequently explored recently. This may be due to their unsatisfactory flexibility, mechanical robustness, the complexity of the production process. An early example includes work by Lee et al. in 2009, where they developed an OPV that could achieve a PCE of 3.27% [141]. Later, Chuangchote et al. proposed fiber-based polymer photovoltaic cell designs in 2011 (see Figure 12), and explored various cell designs in a flat configuration; however, the greatest PCE reported was 0.11% [140].

**Figure 13:**
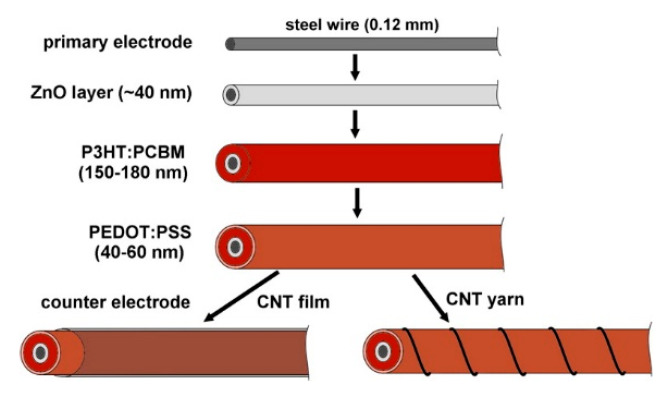
Diagram illustrating the fabrication process and structure of the fiber-shaped solar cells developed by Liu et al. Reprinted with permission from [142]. Copyright 2012 American Chemical Society.

Liu et al. created polymer based cells in 2012 [142] with PCEs as high as 2.3%. Bendability was demonstrated, with the device showing little change when bent up to 90°. The long-term stability was explored, seeing a drop in efficiency to 1.77% after five days and then stabilizing at 1.6–1.7% PCE up to day 14 (stored in inert gas). The paper also detailed a cell design where a CNT yarn was used as a counter electrode instead of a CNT film, showing a PCE of 2.11%. This device showed little change in performance when bent up to 180°. A diagram illustrating the designs used is shown in Figure 13.

In 2012, Zhang et al. proposed a cell design based on CdSe grown on a Ti wire [143]. The cells showed a PCE of 1–2%. It was reported that the device remained stable when rotated or bent. A CuInSe_2_-based cell from 2012 could achieve a PCE of 2.31% [144], and was highly flexible showing only a small reduction in PCE when bent up to 360°. The device was also shown to be stable over a period of 600 h.

### 8.2. 1D Photovoltatic Devices Conducted Using Polymeric Fibers as a Core

With the aim of creating a 1D PV device with improved mechanical robustness and conformability, studies have investigated PV fibers using a polymer core. In 2008 O’Connor and co-workers used a polyimide-coated silica fiber as the core of an OPV fiber, which had a PCE of 0.5% [145]. A DSSC fiber was realized by Toivola et al. in 2009, where both polymethyl methacrylate and glass optical fibers were explored for use as a core material. These PV fibers did not generate a notable amount of power (PCE <0.1% for both the polymethyl methacrylate and glass optical fiber designs) [146]. Bedeloglu et al. fabricated a polymer PV fiber in 2010 starting with a polypropylene fiber core, this only generated a PCE of 0.021% [147].

A stretchable DSSC was presented by Yang et al. in 2014, where a CNT fiber tape was helically wrapped around a rubber fiber to be used as the elastic counter electrode [148]. A helical Ti/TiO_2_ working electrode adsorbed with the dye-sensitizer was wrapped around the elastic counter electrode. This construction was highly conformable and was able to generate a PCE of 7.13% which was maintained during stretching. There was an insignificant change in PCE after 20 stretching cycles of 30%.

### 8.3. Discussion on Photovoltaic Wires, Fibers, and Yarns

The key information regarding photovoltatic wires, fibers, and yarns is summarised in Table 2.

As with devices constructed with PV films or coatings, flexability is a commonly reported property. Many of these works also demonstrated the durability of the PV devices to multiple bend cycles. Further, the ability to weave some of these devices has been reported. Beyond bending, He et al. presented a twistable device that was also 3D deformable [137], and Yang et al. presented a stretchable device [148]. The long term stability of these devices has only been reported in a few cases. PCEs for these types of devices ranged from being negiligable to 10% for the DSSCs produced by Fu et al. [135].

The textile performance of fiber- and tape-based PV devices needs to be explored in planar form (i.e., fabric form) in order to truly establish their feasibility for wearable applications. Their mechanically robustness, aesthetics, and wash durability in fabric structures need to be fully examined. The long-term stability of the devices must also be understood. In principle, these PV fiber devices can be used to make fabrics that exhibit some desirable textile characteristics, such as air permeability and shear deformability; however, their appearance, surface texture, and flexibility may be significantly different from that of textile fabrics made of conventional textile yarns intended for wearable applications.

## 9. Textiles Woven from Photovoltatic Fibers

Flexible planar PV devices can be created by integrating fibers, yarns or tapes containing PV materials into a woven structure.

Fabrics woven with yarns or tapes made from PV materials benefit from the inherent flexibility and conformability rendered by the interlacing of yarns or narrow-tapes in the woven structure, where the individual elements of the weave are free to undergo relative deformations due to external stresses, even if the individual elements are not stretchable. A woven textile with yarns or tapes made from PV materials would also allow the structure to permeate air and moisture, which is critical for a wearable application.

Most of the woven fabrics in the literature created PV modules by combining separate working electrode and counter electrode fibers within the weave structure. Here the interlacing points within the woven structure provided the electrical interconnects to complete the fully functioning PV cell. Another approach is to use fully functional PV wires or yarns with both the electrodes (working electrode and counter electrode) incorporated within the same strand.

In most cases, these woven devices did not report power densities based on the total planar area over which the PV wires were distributed, but instead power densities based on the area covered by the PV elements (wires or narrow tapes). It is important to note that a realistic comparison of such woven devices can only be made with the power densities where the whole fabric area was considered.

### 9.1. Using Interlacing Points to Complete the Photovoltatic Device

An early example of a woven PV fabric was reported in 2012, where a Cadmium selenide (CdSe) coated Ti wire working electrode was interlaced with a set of CNT yarn counter-electrodes [149]. This device demonstrated how a photovoltaic planar structure can be fabricated by using interlacing points to create electrical contacts. The coated wires had a PCE of 1.24%, which was lower than the same coated wire twisted with a CNT counter electrode (PCE of 2.9%; included in Table 2). This was attributed to the comparatively low number of contact points, and to the contact pressure between the two electrodes in the woven structure.

**Figure 14:**
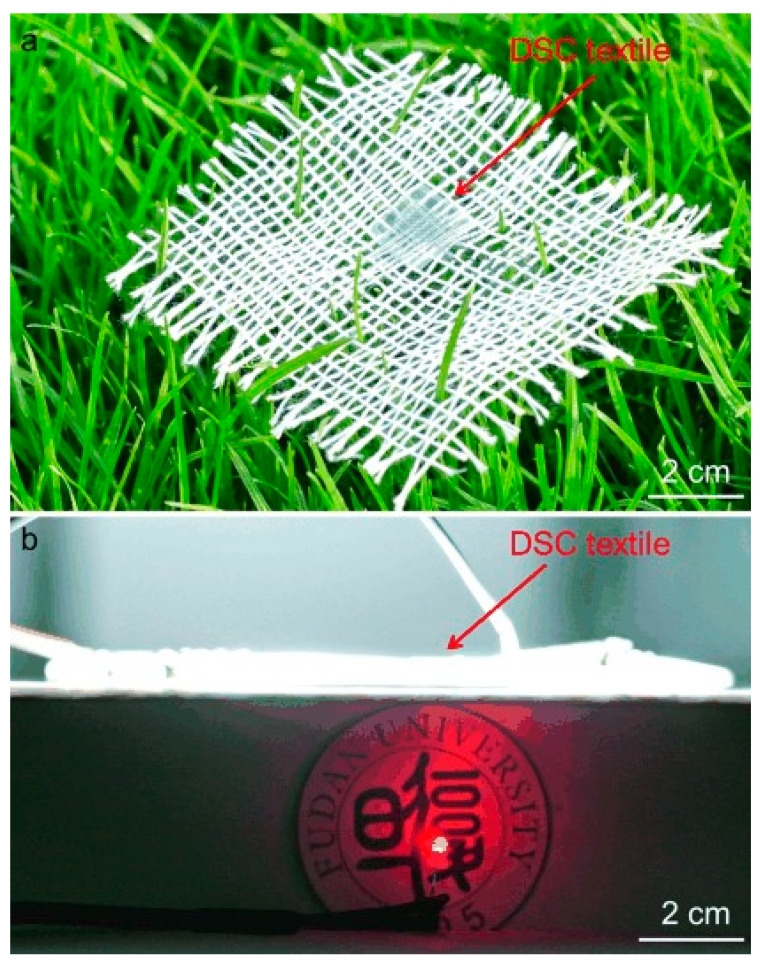
Photographs of the DSSC developed by Pan et al. (**a**) The DSSC. (**b**) The DSSC lighting up an LED. Reprinted from [150] with permission from Wiley.

In 2014, Pan et al. prepared woven mesh electrodes which were stacked together to create a DSSC fabric structure (see Figure 14 for an image of the device), where the stacked electrodes were sealed and injected with an electrolyte to realize a DSSC which exhibited a PCE of 3.10% using a liquid electrolyte, or 2.1% when using a solid electrolyte [150]. Bending of the device (when made using a liquid electrolyte) showed little effect on performance, and when tested over 100 bending cycles, a change of less than 10% of the initial PCE was observed. The device also worked when under shear deformation, a property seldom reported for E-textiles in the literature. The performance of the DSSC that used a solid electrolyte varied by less than 5% after 100 bending cycles. The device was also highly stable, showing a less than a 6% drop in the original PCE after being exposed to air for 300 h. The PCE of the device showed less dependency on the incident angle of the light in comparison to the planar devices; this behavior is desirable for mobile applications, where the incident light angle is unpredictable. The reason for this uniformity in performance was due to the circular cross sections of the electrodes and the spacing between the adjacent electrodes in the same plane of the fabric; this spacing minimized the shading effect from nearby electrodes at higher incident angles.

In an effort to improve the textile appearance and feel of PV woven textiles, a few attempts to weave PV fiber devices together with conventional textile yarns have been reported in the literature [151,152,153]. In 2015, Zhang et al. demonstrated a PV woven fabric by interlacing all-solid-state co-axial DSSC working electrodes (weft yarns) with metal coated polymer counter electrode (warp yarns) [152]. The functioning solar cell was completed by using the warp-weft interlacing points for the electrical connections between the two electrodes. A single wire solar cell unit was reported to have a PCE of 1.3%, which is lower than some core-sheath type DSSC fibers. Mechanical stress analysis of the PV elements within the woven structure was conducted and the effect of mechanical stress on the PV performance was studied. Furthermore, the effect of the counter electrode (warp) density and the woven pattern (plain, twill or satin) on the PV performance was analysed. The warp densities and the weave structure which realised the highest number of interlacing points (or the contact points between the warp and weft) yielded the best PCEs. Bending tests were conducted at different angles, and no significant change in performance was reported. Cyclic bend testing showed with no significant change in performance after 500 cycles. Fabrics woven using the aforementioned PV fibers and wool yarns were also presented and had a normal textile appearence, and while the energy harvesting capability of the wool blended fabric was not investigated, it was shown to power a digital calculator. The device was also shown to be reasonably stable after 60 days when stored in dry conditions. The same DSSC wire structure was utilized by the team to prepare an all-solid-state textile woven device to simultaneously harvest and store solar energy, which had a PCE of ~1%; however it was not clear whether this PCE calculation was based on the fabric area or the wire area [151]. The researchers reported that the device had a stable performance after two months of exposure to ambient conditions, and after 100 bending cycles of 120°. A similar woven textile that can harvest solar and mechanical energy simultaneously [154] has also been reported. The fabric generated a power density of 25 μW/cm^2^ when worn by a human walking in daylight (light intensity = 80 mW/cm^2^).

Liu et al. reported a woven PV structure in 2018, created by weaving OPV working electrode wires as a weft with counter electrodes as a warp [153]. This work successfully demonstrated the weaving of fiber-shaped PV structures using an industry standard weaving loom, which was a key milestone for textile PV devices. This fabric also employed an interlacing technique to achieve electrical interconnections between the working electrodes and counter electrode wires. The resultant woven fabric exhibited a PCE of 1.62% (in wire form). The device was stable showing ~10% degradation in performance after 15 days exposed to air. The fabric showed a less than 15% reduction in performance after bending up to 80° and exhibited no deterioration of performance after 1000 20° bending cycles and 1000 180° twisting cycles, indicating excellent stability under mechanical deformation. The fabric was very similar to a regular woven fabric in terms of appearance. The device is claimed to be breathable. The device was also demonstrated powering a digital watch. Ultimately the study demonstrated a PV device that possessed many desirable textile properties; however, the PCE was still low compared to coated or laminated textile PV devices. Increasing the density of the working electrodes would increase the PCE of the fabric; however, this would have significant implications for the conformability and softness of the fabric.

### 9.2. Weaving Photovoltaic Ribbons or Tapes

One approach to improve the PCE of woven solar E-textiles is to replace the PV wires with pre-fabricated PV ribbons (also referred to as tapes) [26,155,156,157]. These ribbons were either complete PV devices or constituted some of the active PV layers. PV ribbons have a larger active area and can cover a significantly larger proportion of the available planar surface compared to PV wires. However, PV ribbons can also significantly alter the conformability and appearance of the final fabric depending on the stiffness of a single ribbon and the number of ribbons per unit length within the fabric.

Yun et al. fabricated a woven PV device in 2015 by inserting DSSC-type PV ribbons using a conventional weaving machinery, with the device exhibiting a PCE of 2.63% [156]. These flexible ribbons maintained a performance of 85% of their initial PCE when bent with a 3 cm radius of curvature. In 2015, Krebs and Hösel employed pre-fabricated OPV tapes (~2 cm wide) to weave a large area (25 × 25 cm^2^) woven solar cell textile [155]. The solar tapes woven into the structure consisted of 16 serially connected organic solar cells with a PCE of 1%.

A ribbon-shaped hybrid PV device that could harvest and store solar energy was reported by Li et al. in 2016 [26]. The ribbon was prepared by stacking a perovskite flexible PV film onto a thin film electrochemical supercapacitor, with the top electrode of the supercapacitor also acting as the counter electrode of the PV device. The perovskite PV film generated an impressive 10.41% PCE (under 1 Sun intensity). The cells were bendable, showing a reduction in initial PCE of 10% after 100 bending cycles of 120°. A 10% reduction from the initial PCE was also observed when the laminated cells were exposed to air for ten days. These ribbons could be inserted into a woven structure to demonstrate a wearable energy device; however, this did not resemble a textile material due to large widths (>1 cm) of the tapes used in the work.

A textile-based solar energy harvesting and storage system was created by Kuhlmann et al. in 2018. The device was created by weaving flexible CIGS thin-film PV cells (44 mm wide) with conventional textile yarns and attaching commercially available flexible batteries (solid-state lithium batteries having a thickness of 0.4 mm) [157]. Krebs and Hösel have argued that woven PV tapes are one of the most technically feasible and commercially viable stratergies for incorporating PV functionality with textiles [155]; however, it is doubtful whether such devices are acceptable for use in future wearables intended for day-to-day use, mainly due their lack of normalcy.

### 9.3. Discussion on Textiles Woven from PV Fibers, Ribbons or Tapes

The key information regarding textiles woven using PV fibers, ribbons or tapes are summarised in Table 3.

Flexibility was shown for PV textiles woven using PV fibers, ribbons, and tapes. Many examples in the literature demonstrate the durability of the textiles to repeated bending cycles. Work by Liu et al. also showed twist and twist-durabilty, and Pan et al. showed that the textile had shear behaviour, a crticial factor for a normal textile feel. Given the inherent porous structure, this type of device would also be breathable and would exhibit some moisture transfer characteristics. However, in some cases, the need to fully encapsulate the device will hinder the permeability and flexibility of the final product. This will also alter the appearence.

The long term stabilty of some devices have been reported, with two DSSCs shown to be stable after about two months [151,152]. PCEs reported for these devices vary, with the highest PCE being reported by Li et al. for their Pervoskite device, with an impressive PCE of 10.41% [139].

Further testing is still required to fully understand the viability of this type of PV device for wearable garments. For example, the electrical interconnections achieved by the interlacing points for some devices may not by reliable during the deformations undergone by the resultant garments, and these interconnections could deteriorate during washing and wearing.

## 10. Non-Woven Photovoltaic Textiles

Non-woven textile structures such as electrospun nano-fiber webs or fiber membranes have also been investigated as a substrate for textile PV devices [158,159,160].

In 2010, Sundarrajan et al. realised an electrospun polymeric PV web [158]. The active polymer layer of the PV web was sandwiched between transparent conductive electrode films. A notable PCE was not reported for the device.

A DSSC based on a PET membrane (as the medium for liquid electrolyte) was developed by Sun et al. in 2016 [159], which reported a PCE of 10.2%. The stability of these cells was investigated and monitored over four weeks at atmospheric conditions and 60 °C, with most varients of these cells maintaining 70–75% of their initial PCE after this time. The photoanode and counterelectrodes used in this device were monolithic and non-conformable, hence the device did not possess qualtities that were desirable for wearable applications.

A DSSC PV device with textile-based counter electrodes was investigated by Junger et al. in 2018 [160]. The PV device was conformable, however the PCE achieved was not noteworthy.

## 11. Discussion about Solar Energy Harvesting Textiles

This review has collated various literature sources where PV has been incorperated with textiles to provide energy harvesting capabilities. The review has highlighted the various means by which PV can be combined with textiles, and when reported, has highlighted the textile characteristics of these devices.

### 11.1. Textile Properties of Photovoltaic Textiles

While capable of generating significant amounts of power, attaching flexible solar panels onto garments will significantly affect the look and feel of the textile. While these asthetics may be desirable for some niche applications, such as for fashion prototypes, this is not favorable for most consumer applications. By including solar panels on the textile in a dispersed pattern some flexibility and drapeability can be maintained, which may be acceptable for heavy outwear; however this would likely be uncomfotable if applied to a garment that is intended to be worn close to the skin. Similarly, the presence of attached solar panels will negatively affect the heat and moisture transfer characteristics of the garment. From the available literature, the durablility of garments of this type is unclear.

Solar cell arrays can lack a normal textile appearance, and while being flexible, do not necessarily exhibit other textile properties such as shear deformability, which is key for drapability. Embedding arrays of solar cells within yarns was the only example in the literature of solar cell array-based fabric that maintained a normal textile appearance and textile properties (i.e., drape, washability) [101].

PV films or coatings can be applied onto textiles; however, in order to achieve long term stability and compatibility with water, the devices have to undergo an encapsulation step using a thin film or a flexible coating (which are sometimes elastic) with excellent barrier properties. Regardless of the approach, the use of an encapsulation step results in solar E-textiles that are non-permeable and that have a non-porous structure. Despite being inherently flexible, when applied onto large area textiles (which are inherently porous due to the openness of woven or knitted structures), these PV materials inhibit the air and moisture permeability of the base textile, which is critical for the comfort of the wearer, especially during warm and humid conditions. These systems also affect the appearance and feel of the textile. Limited information is presented on the durability of the textiles for wearable applications, however Jinno et al. demonstrated the washability of their OPV film-based device [104]. Jeong et al., in 2019, also demonstrated the washability and water compatibility for their OPV device, as well as resilience to bend testing [115]. While these two devices showed great promise in achieving wash durable PVs, the washability and water compatibility tests employed in these studies were mild, and far from the vigorous hydro-mechanical, thermal and chemical processes undergone by regular clothing in a domestic machine washing.

There has been a wide range of work investigating fiber/wire/tape-shaped PV devices intended to be transformed into planar form (e.g., a woven textile); however, many have only been demonstrated in fiber/wire form. Tests of this type of PV device have largely been limited to flexibility and durability to repeated bending cycles.

Similarly, fabrics woven with PV-coated wires or flexible PV tapes are often only tested for their flexibility. Given their woven nature, these PV devices will have a porous structure and should be breathable and exhibit moisture transfer characteristics. The structure may also give these devices a more normal textile behavior (i.e., shear). Some PV devices of this type will not have a normal textile appearance; the OPV-based fabric created by Liu et al. is one example of a solar E-textile that maintained the aesthetics of a regular fabric [153].

From the existing literature, it is evident that most of the work on solar E-textiles to date has not focused on the textile aspects of the E-textile. In most of the literature, flexibility is considered the prime requirement for wearability; however, in reality it is often a combination of many facets such as softness, shear behavior (this is key to three-dimensional conformability), appearance, air/moisture permeability, heat-transfer characteristics and wash durability. There exists a gap in knowledge on how these features can be achieved, while maintaining satisfactory power conversion efficiencies. The physical durability of the textile is also highly important for a wearable application, however beyond flexibility/bending, few examples of durability testing have been presented. Table 4 summarizes examples of solar E-textile where considerations beyond flexibility have been reported.

It is clear that further extensive testing of solar E-textiles is necessary, both to characterize properties and to ascertain their durability. The lack of testing for textile properties to date is likely in part due to the limited scale of production of these PV devices. Certain types of textile testing may prove critical for some solar E-textile designs, for example, abrasion testing may wear away the thin protective encapsulation necessary for many of the proposed designs, significantly limiting lifetime. Damaging the protective encapsulant may also lead to the wearer being exposed to the constituent materials of the solar cell. This may be problematic for lead-containing perovskite solar cells, which may pose a safety risk.

The stability of many solar E-textiles also needs to be improved. If used in a commercial product, the solar elements will be expected to remain functional for many years. This may influence the technologies and materials adopted moving forwards. While exhibiting good PCEs, even state-of-the-art perovskite solar cells (not designed not textile applications) only retain their initial performance for several months [161]. This is in part due to perovskite’s instability to moisture and oxygen. Perovskite also has poor thermal stability, and degrades when exposed to light.

Table 5 summarizes details about the different methods used to create solar E-textiles.

### 11.2. Suitability for Powering E-Textile Sensing Systems

The energy harvesting capability of a solar E-textile is the other key consideration of its suitability for powering an E-textile device. Different E-textile devices possess different energy needs depending on their function and use case; however, the power requirements for wearable devices typically range between tens and hundreds of milliwatts [162], with most mobile devices, such as mobile phones, smart watches and fitness trackers, requiring 50 mW to 1000 mW during normal use [163].

While some solar E-textiles shown in the literature exhibit a poor PCE, many have a PCE of over 1%, and PCEs exceeding 10% have also been reported [159]. Textiles and garments are large structures providing a significant area onto which PV technology can be integrated. Therefore, despite lower PCEs than conventional solar cells, the inefficiency in power conversion of the textile PV devices can be partially mitigated by harvesting energy over a large area. One Sun intensity is 100 mW/cm^2^; therefore, a solar E-textile with a conservative PCE of 1% covering an area of 1000 cm^2^, would be able to power a device that requires 1 W. An active area of this size could easily be fit onto the back of a conventional T-shirt. This scenario, however, assumes ideal conditions where the light intensity continuously reaching the solar E-textile is 100 mW/cm^2^, which would not be true in most cases given shading, cloud cover, or change in the angle of incident light. Due to a potentially inconsistent intensity of light reaching the solar E-textiles, most systems would likely employ energy storage as part of their power management electronics.

Fully assessing the suitability of different solar E-textiles for powering E-textile devices is difficult given the widely varying power requirements, intended application (as this will influence the intensity of light reaching the device), and garment design parameters (potentially limiting the size of the active area). Despite this, solar E-textiles remain the best candidate for harvesting energy levels sufficient to power wearable devices.

### 11.3. Future Perspectives for Solar Energy Harvesting Textiles for Wearable Applications

This review has identified the key methods explored in the literature to intergrate solar energy harvesting functionality into textiles. Beyond attaching solar cells to outerwear, most integration technologies are far away from being ready as commerical devices. Given the level of maturity of these technolgies compared to other devices in the E-textiles field, it is likely that the trend of using batteries to power E-textiles will continue while the exciting innovations described in this review evolve further.

The future advancement and, ultimately, the adoption of solar E-textiles will require the textile characteristics to be fully quantified and the durability of the devices for use as textile garments to be understood. This will be challenging given the lack of standards currently available for the testing of E-textiles (currently only BS EN 16812:2016 exists). Many devices, in their present form, may not possess the desired properties or robustness, and therefore further development will be needed; however, this is difficult to conclusively comment on without testing data. The stability of many solar E-textiles will also need to be improved to allow for the long term use demanded of a product. The processes to manufacture these textile solar cells at a large scale will also be nessisary.

Finally, power management will need to be addressed. The output from a solar E-textile will not be continuous due to the movement of the wearer, and the position of the Sun. A further hurdle to the adoption of solar E-textiles will be the creation of power management electronics that can also be discretely integrated within a textile.

## Figures and Tables

**Table 1:** Summary of key information regarding the reported properites of various photovoltatic films and coatings used on textiles. Please note that PCE (%) are the values given and do not account for any differences in how the PCE may have been calculated between works. ‘Likely flexible’ has been stated when the flexability of the device is not explictily commented upon or shown.

	Type of SC	PCE (%)	Mechanical Deformability	Mechanical Durability	Washability	Stability	Other
Krebs et al. [103]	OPV	0.0014	Flexible			Substantial degradation after 2 h	
Bedeloglu et al. [110]	OPV	0.33	Flexible				
Lipomi et al. [111]	OPV	2	Flexible, stretchable				
Kaltenbrunner et al. [112]	OPV	4	Flexible, stretchable, 3D deformation (drape)	Cyclic compression tested to 22 cycles.			
Kylberg et al. [113]	OPV	2.2	Flexible				
Lee et al. [105]	OPV	1.8	Likely flexible				
Arumugam et al. [114]	OPV	0.02	Flexible	Bend and cyclic bend tests led to loss of functionality.			
Jinno et al. [104]	OPV	7.9	Flexible, stretchable	Cyclic compression testing to 20 cycles.	Compatible with water, washable under mild conditions.	46% decrease in PCE after 1000 h	
Jeong et al. [115]	OPV	7.26	Flexible	1000 bend testing cycles		No notable degradation after 30 days.	
Du et al. [102]	DSSC	3.93	Flexible				
Xu et al. [116]	DSSC	3.3	Flexible				
Xu et al. [117]	DSSC	3.83	Likely flexible				
Yun et al. [118]	DSSC	4.16	Flexible	Bend testing to 2000 cycles.			
Opwis et al. [119]	DSSC	1.1	Flexible			Stable after seven week.	Performance at different operating temperatures reported.
Liu et al. [106]	DSSC	2.78					
Yun et al. [120]	DSSC	1.7	Flexible	Bend testing to 2000 cycles.			
Song et al. [122]	DSSC	5.08	Flexible				
Knittel et al. [123]	CIGS	8	Flexible				
Plentz et al. [124]	a-Si	1.41	Likely flexible				
Lam et al. [108]	Perovskite	15	Likely flexible		Compatible with water	Maintained 70% initial PCE after 425 h	
Jung et al. [109]	Perovskite	5.7	Flexible			Maintained 83% initial PCE after 300 h	

**Table 2:** Summary of key information regarding the reported properites of various photovoltatic wires, fibers, and yarns. Please note that PCE (%) are the values given and do not account for any differences in how the PCE may have been calculated between works.

	Type of SC	PCE (%)	Mechanical Deformability	Mechanical Durability	Washability	Stability	Other
Ramier et al. [125]	DSSC	Nominal		Strain and bending tested, however poor mechanical robustness reported.			
Lv et al. [126]	DSSC	5.41	Rigid				
Yang et al. [127]	DSSC	8.45	Flexible, high mechanical strength			Claimed to have good stability	
Liang et al. [128]	DSSC	9.1	Flexible	Bend testing conducted with different bending angles			
Chen et al. [129]	DSSC	2.94	Flexible			Claimed to have good stability	Shown woven into a textile
Chen et al. [132]	DSSC	4.6	Flexible				
Velten et al. [130]	DSSC	3.4	Likely flexible				
Yan et al. [131]	DSSC	6.24	Flexible			Stability shown in earlier work	Temperature stability shown
Zhang et al. [133]	DSSC	4.85	Flexible	500 bending cycles		Limited lifetime	
Uddin et al. [134]	DSSC	2.57	Flexible			Stable over 50 h	Temperature stability shown
Fu et al. [135]	DSSC	10	Flexible	2000 bending cycles			Shown powering a pedometer
Qiu et al. [136]	Perovskite	3.3	Flexible	50 bending cycles			Shown woven into a textile
He et al. [137]	Perovskite	2.61	Flexible, twistable, 3D deformable	200 bending cycles, 100 twisting cycles			Shown woven into a textile
Hu et al. [138]	Perovskite	5.3					
Li et al. [139]	Perovskite	3.03	Flexible	1000 bending cycles		89% of initial performance after 96 h	
Chuangchote et al. [140]	OPV	0.11					
Lee et al. [141]	OPV	3.27	Flexible				
Liu et al. [142]	OPV	2.3	Flexible			PCE of 1.7% after 14 days	
Zhang et al. [143]	Flexible inorganic	2	Flexible				
Zhang et al. [144]	Flexible inorganic	2.31	Flexible			Stable when tested for 600 h	
O’Conner et al. [145]	OPV	0.5					
Toivola et al. [146]	DSSC	<0.1					
Bedeloglu et al. [147]	OPV	0.021	Flexible				
Yang et al. [148]	DSSC	7.13	Flexible, stretchable	20 stretch cycles at 30% stretch			
Zhang et al. [149]	Inorganic	2.9					

**Table 3:** Summary of key information regarding the reported properites of various textile woven using photovoltatic fibers, ribbons, or tapes. Please note that PCE (%) are the values given and do not account for any differences in how the PCE may have been calculated between works.

	Type of SC	PCE (%)	Mechanical Deformability	Mechanical Durability	Washability	Stability	Other
Zhang et al. [149]	Inorganic	1.24	Flexible				Shown as a woven device
Pan et al. [150]	DSSC	3.67	Flexible, shear behavior	100 bending cycles			Shown as a woven device, shown powering an LED
Pan et al. [150]	DSSC (solid electrolyte example)	2.1	Flexible	100 bending cycles		Stable when tested for 300 h in air	Shown as a woven device
Zhang et al. [152]	DSSC	1.3	Flexible	500 bending cycles		Stable after 60 days	Shown as a woven device, normal textile appearance, shown powering a calculator
Chai et al. [151]	DSSC	1	Flexible	100 bending cycles		Stable after two months	Shown as a woven device
Chen et al. [154]	DSSC		Flexible				Shown as a woven device, foldable, hybrid energy harvesting device
Liu et al. [153]	OPV	1.62	Flexible, twistable	1000 bending cycles, 1000 twisting cycles		Stable after 15 days (10% reduction in initial PCE)	Shown as a woven device, normal textile appearance
Yun et al. [156]	DSSC	2.63	Flexible				Shown as a woven device
Krebs and Hösel [155]	OPV	1	Flexible				Shown as a woven device
Li et al. [26]	Perovskite	10.41	Flexible	100 bending cycles		Stable after 10 days (10% reduction in initial PCE)	Shown as a woven device
Kuhlmann et al. [157]	CIGS		Likely flexible				Shown as a woven device

**Table 4:** Examples of solar E-textiles were textile properties other than flexibility and durability to repeated bending have been reported. Please note that PCE (%) are the values given and do not account for any differences in how the PCE may have been calculated between works.

	Type of SC	PCE (%)	Mechanical Deformability	Mechanical Durability	Washability	Other
Satharasinghe et al. [101]	c-Si array	2.15	Shear behavior, drapeable	Abrasion testing to 6000 abrasion cycles	Retained ~90% of their original power output after 15 machine wash cycles	Woven device, shown powering various devices
Lipomi et al. [111]	OPV film	2	Stretchable			
Kaltenbrunner et al. [112]	OPV film	4	Stretchable, 3D deformation (drape)	Cyclic compression tested to 22 cycles.		
Jinno et al. [104]	OPV film	7.9	Stretchable	Cyclic compression testing to 20 cycles.	Compatible with water, washable under mild conditions.	
Lam et al. [108]	Perovskite laminate	15			Compatible with water	
He et al. [137]	Perovskite fiber	2.61	Twistable, 3D deformable	100 twisting cycles		Shown woven into a textile
Yang et al. [148]	DSSC fiber	7.13	Stretchable	20 stretch cycles at 30% stretch		
Pan et al. [150]	DSSC woven textile	3.67	Shear behavior			Shown as a woven device, shown powering an LED
Liu et al. [153]	OPV woven textile	1.62	Twistable	1000 twisting cycles		Shown as a woven device, normal textile appearance

**Table 5:** Summary of key details reported on solar E-textiles. Not all devices will have these properties or PCEs. Please note that PCE (%) are the values given and do not account for any differences in how the PCE may have been calculated between works.

	Maximum Reported PCE	Mechanical Deformability	Mechanical Durability	Washability	Maximum Stability Reported	Other
Integrating flexible solar panels with textiles	Dependent on attached cell	Flexible	Likely durable to some wear and use	Not reported	The a-Si type of cells used are typically stable	
Solar cell arrays	14.9	Flexible, shear behavior, drapeable	Abrasion testing to 6000 abrasion cycles	Water compatible, can be machine washed	c-Si type cells are typically stable	Breathable
PV films and coating applied to textiles	7.9	Flexible, stretchable	Cyclic bend testing, cyclic compression testing	Water compatibility reported, can be washed under mild conditions	No degradation after 30 days reported	
Photovoltaic wires, fibers, and yarns	10	Flexible, twistable, stretchable, 3D deformable	Cyclic bend testing, cyclic stretching, cyclic twisting		Stable after 25 days reported	
Textiles woven from photovoltatic fibers	10.41	Flexible, twistable, shear behavior	Cyclic bending, cyclic twisting		Stable after 2 months	Breathable
Non-woven photovoltaic textiles	10.2					
